# Latent infection of human cytomegalovirus is associated with the development of gastric cancer

**DOI:** 10.3892/ol.2014.2148

**Published:** 2014-05-16

**Authors:** JINJI JIN, CHANGYUAN HU, PENGFEI WANG, JING CHEN, TIANTIAN WU, WENJING CHEN, LULU YE, GUANGBAO ZHU, LIFANG ZHANG, XIANGYANG XUE, XIAN SHEN

**Affiliations:** 1 Department of General Surgery, The First Affiliated Hospital of Wenzhou Medical University, Wenzhou, Zhejiang 325000, P.R. China; 2 Department of Rheumatology, The First Affiliated Hospital of Wenzhou Medical University, Wenzhou, Zhejiang 325000, P.R. China; 3 Department of Microbiology and Immunology, Wenzhou Medical University, Wenzhou, Zhejiang 325035, P.R. China

**Keywords:** human cytomegalovirus, infection, gastric cancer, UL133-UL138 locus

## Abstract

The worldwide contagion, human cytomegalovirus (HCMV), may cause a series of disorders in infected individuals. The aim of the present study was to investigate whether HCMV infection is associated with the development of gastric cancer. In this study, the positive expression of unique long (UL)133-UL138 and immediate-early (IE)1 genes, which are associated with viral latency and replication, respectively, were detected using nested polymerase chain reaction. A χ^2^ test and logistic regression analysis were performed to further investigate the preliminary data. The data indicated that the positive rate of UL133, UL135 and UL136 expression in cancer tissues was higher than that in paired normal tissues (P=0.01, 0.027 and 0.013, respectively). However, no significant differences were identified in the UL133–138 locus and IE1 gene when associated with clinicopathological features. Furthermore, seven infection patterns were identified, with the UL133 + UL138 infection pattern representing the largest proportion in the cancer (60.34%) and normal tissues (42.11%). In conclusion, it is possible that the UL133–UL138 locus is important in the occurrence of gastric cancer. The mechanism by which UL133–UL138 locus expression differs in human gastric cancer requires further investigation.

## Introduction

Gastric cancer is one of the most common malignant tumors of the gastrointestinal tract, exhibiting high morbidity and mortality rates, particularly in Northeast Asia, including China, Japan and South Korea (1,2). The pathogenesis of gastric cancer remains unclear, however, social-economic environment, lifestyle, nutrition, education, smoking and *Helicobacter pylori* infection are all associated with its occurrence (3–6).

Human cytomegalovirus (HCMV) is a ubiquitous β-herpes virus that may cause the infection of multiple cell types in human hosts. The virus persists in 30–100% of the population worldwide, particularly in certain areas of Africa and Asia, via three different infection modes: Acute, persistent and latent infections (7,8). Asymptomatic infection, caused by latent state HCMV in healthy individuals, may increase the risk of atherosclerosis and age-related immune senescence (9,10). Furthermore, severe or acute disease may be induced in immunocompromised hosts, including acquired immunodeficiency syndrome patients and transplant recipients, due to the reactivation of latent HCMV (11,12). An increased number of cases of gastrointestinal diseases caused by HCMV infection have been reported, including ulcerative colitis and esophageal ulcers (13,14). In addition, emerging evidence has indicated that HCMV infection may be associated with human malignancies, including colon and prostate cancer (15,16). However, the association between gastric cancer and HCMV remains unclear.

The HCMV genome encodes >200 predicted open reading frames (ORFs) and is comprised of unique long (UL), unique short and other repeated sequences (8,17). The locus spanning UL133–UL138, but particularly UL138, within the ULb’ region is considered to be important for viral latency (18–21). The reactivation of latent virus (viral replication) may be estimated by detecting the expression of the immediate-early (IE) gene (22,23). In the present study, the expression of UL133–UL138 and IE1 (UL123) were investigated in gastric cancer and corresponding normal tissues using nested polymerase chain reaction (PCR), and the clinical association between gastric cancer and HCMV infection was evaluated.

## Materials and methods

### Patients and specimens

The paired tissue samples used in the present study consisted of gastric adenocarcinoma and corresponding normal tissues, which were obtained by negative resection margin. The samples were snap-frozen in liquid nitrogen within 30 min of resection and stored for RNA/DNA extraction. All specimens were obtained from patients diagnosed with gastric cancer (n=60) by endoscopic biopsy who underwent surgery at The First Affiliated Hospital of Wenzhou Medical University (Wenzhou, China) between February 2011 and December 2012. No patients received radiation or chemotherapy prior to surgery. The histopathological diagnosis of gastric adenocarcinoma was confirmed following surgery by the Department of Pathology according to the criteria of the World Health Organization (24). Table I shows the clinicopathological features of the cancer patients according to the National Comprehensive Cancer Network (2012) guidelines (25). Informed written consent was obtained from all patients and the study was approved by the Human Research Ethics Committee of The First Affiliated Hospital of Wenzhou Medical University.

### Homology and similarity analysis

A total of 18 HCMV genomes were found and downloaded from the National Center for Biotechnology Information GenBank (http://www.ncbi.nlm.nih.gov/) and also from the University College London virus database (http://www.biochem.ucl.ac.uk/bsm/virus_database/VIDA_table_herpesviridae_cg.html). The similarity of each UL133–UL138 and IE1 coding sequence was examined using Basic Local Alignment Search Tool (http://blast.ncbi.nlm.nih.gov/Blast.cgi) and aligned by clustalx2 (ftp://ftp.ebi.ac.uk/pub/software/clustalw2/). The corresponding coding sequences in the varying strains were then used for homology analysis and specific primer design.

### RNA isolation and nested PCR

Total RNA was extracted from frozen tissue specimens using TRIzol reagent (Invitrogen Life Technologies, Carlsbad, CA, USA) according to the manufacturer’s instructions. Next, the first-strand complementary DNA was reverse transcribed using 1 μg total RNA and the reverse transcription kit (Toyobo Co., Ltd., Osaka, Japan) according to the manufacturer’s instructions. The corresponding primers and the conditions of PCR amplification, as performed by the pre-programmed Thermal Cycler (Bio-Rad, Hercules, CA, USA), are listed in Table I. Following amplification, the PCR products were subjected to electrophoresis in 2% agarose gel, stained with ethidium bromide, and images were captured using an ultraviolet light transilluminator (Bio-Rad). The UL133–UL138 and IE1 PCR amplification products were then purified, cloned into a pMD19-T Vector (Takara Bio, Inc., Shiga, Japan) and transformed in *Escherichia coli* DH 5α (Novagen, Merck KGaA, Darmstadt, Germany). Finally, the positive colonies (three monoclones per sample) were sequenced using M13 sequencing primers [Sangon Biotech (Shanghai) Co., Ltd., Shanghai, China] by the 3730xl DNA Analyzer (Applied Biosystems, Carlsbad, CA, USA) to confirm the PCR specificity.

### Statistical analysis

Statistical analyses were performed to investigate the differences in UL133–138 and IE1 expression between specimens using the χ^2^ test, and Fisher’s exact test was used for samples with small sample numbers. Logistic regression analysis was used to assess the effect of those loci in the cancer tissues. P<0.05 was considered to indicate a statistically significant difference and all analyses were performed using SPSS version 16.0 (SPSS, Inc., Chicago, IL, USA).

## Results

### Homological analysis of UL133–138 and IE1 coding sequences in different HCMV strains

To establish nested PCR, firstly the similarities between the UL133–138 and IE1 coding sequences were analyzed in different HCMV isolates. The data indicated that the UL133–138 and IE1 coding sequences exhibited a relatively high similarity among the 18 HCMV strains (data not shown). The homologies of the nucleotide sequences were 94.39±2.02 (range, 91.12–100), 98.57±0.69 (range, 97.52–100), 98.65±0.60 (range, 97.79–100), 97.96±0.95 (range, 96.47–100) and 97.53±1.25 (range, 95.26–100), respectively. Based on these observations, specific primers were designed to detect the expression of these genes in neoplastic and normal gastric tissues.

### Accuracy and specificity of the nested PCR assay

**Figure 1:**
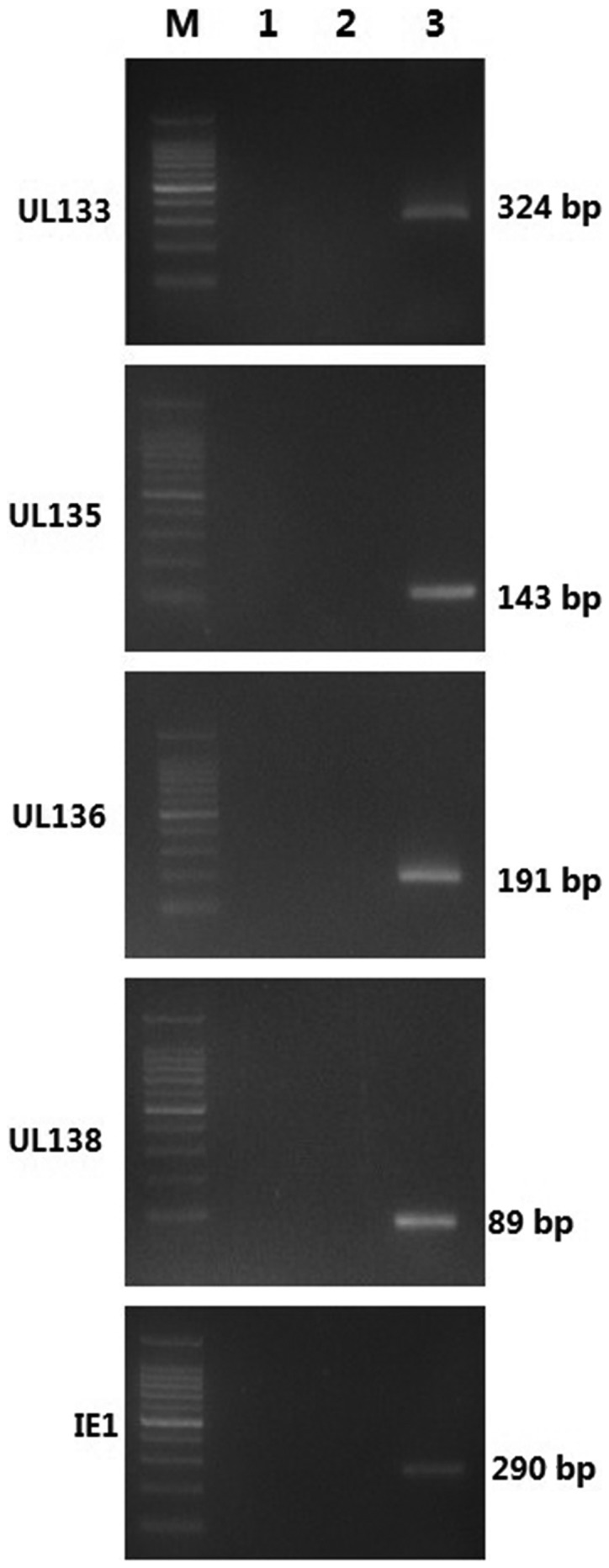
Detection of HCMV UL133–UL138 and IE1 complementary DNA transcripts in tissues. M, molecular marker [100-bp DNA ladder; Lane 1, ddH_2_0 blank control; Lane 2, corresponding RNA control; and Lane 3, corresponding positive cDNA to verify the specificity of the primers; HCMV, human cytomegalovirus.

To assess the accuracy and specificity of nested PCR, a minus-reverse transcription (RT) control was set up, in which RT templates were replaced by water or RNA. As shown in Fig. 1, electrophoresis revealed a single band of UL133–UL138 and IE1 at the appropriate positions (324 bp for UL133, 143 bp for second stage UL135, 191 bp for second stage UL136, 89 bp for second stage UL138 and 290 bp for IE1, respectively). No PCR product was observed in the minus-RT control. Additional sequencing further confirmed the specificity and accuracy of the nested PCR assay.

### UL133–138 and IE1 expression in gastric cancer and adjacent normal gastric tissues

**Figure 2:**
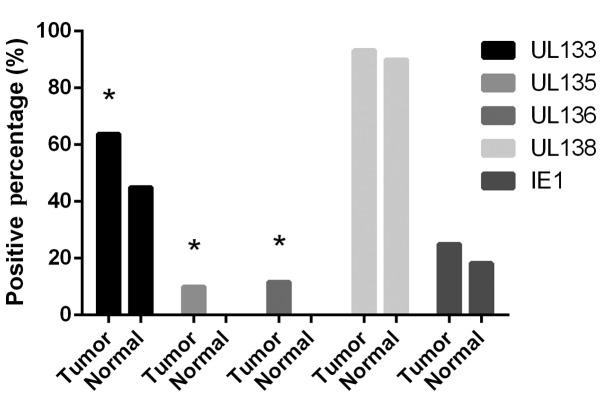
Comparison of the positive rates of UL133–UL138 and IE1 in normal and tumor tissues (^*^P<0.05 vs. normal). The relative expression of UL133–138 and IE1 in the tumor and corresponding non-tumor tissues are presented. A statistically significant difference was identified between UL133, UL135 and UL136, and the positive cases of UL135, whereas only UL136 expression was detected in the gastric cancer tissues. UL, unique long; IE, immediate-early.

The nested PCR method described previously was used to further investigate the expression of IE1 and individual genes in the UL133–UL138 locus in paired neoplastic and normal gastric tissues. As shown in Fig. 2, the detection rate of IE1 was 25.00% (15/60) in the cancer tissues and 18.33% (11/60) in the normal tissues. No significant differences were identified between the malignant and normal tissues (P=0.375). The UL133 expression rate was found to be 68.33% (41/60) in the gastric cancer tissues, which was significantly higher than that in the corresponding normal tissues (45.00%; 27/60; P=0.01). Notably, the expression of UL135 and UL136 was only positive in the gastric cancer samples, in six cases (10%) for UL135 and seven cases (11.67%) for UL136 (P=0.027 and 0.013, respectively). The highest level of expression was detected from the UL138 gene in the tumor and normal tissues. The expression of UL138 in the tumor and corresponding normal tissues was 93.33% (56/60) and 90.00% (54/60), respectively, and no significant difference was identified between the two groups (P=0.509).

### Correlation between the UL133–138 locus and clinical analysis

No significant differences were identified between the expression of UL133, UL135, UL136, UL138 and IE1 and the clinicopathological features of gastric cancer, including pathological differentiation type, tumor-node-metastasis staging, diameter of tumor, and patient age and gender (Table II). Further analysis of the pattern of infection was performed, and as a result, seven infection patterns in the gastric cancer tissues and three patterns in the corresponding normal tissues were summarized (Table III). The pattern of UL138 alone accounted for 18.97% (11/58) of all patterns observed in the cancer tissues, while the same pattern was detected in 52.63% (30/57) of the normal tissues. Furthermore, a statistically significant difference was identified in this pattern transcript in different tissues (P<0.01). The UL133 + UL138 infection pattern occupied the largest proportion, detected in 60.34% (35/58) of the cancer tissues, which was similar to the phenomenon observed in the normal tissues (24/57). However, 69.64% (39/56) of UL138 positive cases were detected in the UL133 transcripts in the gastric cancer tissues, while the ratio in normal tissues was 44.44% (24/54) (P=0.008). The patterns, including UL135 or/and UL136, were found in 17.24% (10/58) of transcripts and were only detected in cancer tissues.

## Discussion

Increasing evidence indicates that HCMV may be associated with certain human malignancies, including cancers of the brain, colon, breast and prostate (15,16,26,27), and rhabdomyosarcoma (28). However, no studies have investigated the association between HCMV and gastric cancer. In addition, similar to other herpes viruses, a key biological property of HCMV is latent infection, during which, the viral genome persists in the absence of the production of an infectious virus and only a subset of viral genes are expressed (29,30). Few studies have explored the status of HCMV infection in cancerous tissues.

Previous studies have indicated that the UL138 ORF is highly conserved in clinical strains and is thus important in latent HCMV infection (31). The UL133–UL138 locus has also been found to suppress viral replication thereby promoting latent infection (20). However, the IE gene is critical for the detection of viral DNA replication, viral reactivation and the differentiation of monocytes to macrophages (22,23). In the present study, it was confirmed that gastric cancer is associated with latent HCMV infection, and that the genes of IE1 and the UL133–UL138 locus are expressed in the epithelium of neoplastic and normal tissues. Furthermore, the detection of the expression of UL133, UL135, UL136 genes, which are associated with HCMV latency, were found to be significantly different in the normal and cancer tissues, whereas no significant difference was identified in the expression of IE1, which correlates with viral replication, between malignant and normal tissues. The transcripts of UL135 and UL136 were only detected in the gastric cancer tissues. Notably, the majority of the positive UL135 and UL136 transcripts were found concentrated in poorly-differentiated tissues, and in the tissue of positive patients at stage III of gastric cancer. These results indicate that UL136 and UL135 are risk factors associated with the development of gastric cancer. In addition, the results confirmed that latent HCMV infection, but not replication infection, may have a close association with the occurrence and development of neoplastic differentiation and the staging progress. However, the low detection rate of transcription that was observed limits further explanations (20).

At present, there is no conclusive evidence that the virus exhibits an oncogenic role, as normal cells do not appear to be transformed following infection (32,33). Numerous studies support the theory that HCMV may be oncomodulatory in the neoplastic process (33–35). This theory suggests that HCMV infection may induce cellular responses that provide favorable conditions for the growth of neoplastic cells. In addition, it has been reported that HCMV infection may affect cell signaling pathways (36), release inflammatory cytokines (37), promote immune evasion (38,39), inhibit cancer cell apoptosis (33), cause DNA mutations and deregulate the cell cycle of infected cells (40,41). Therefore, the combined effects of HCMV genes orchestrated by viral and cellular mechanisms during HCMV infection may present a significant mechanism in the neoplastic process.

Previous studies have reported that in the UL133–138 locus, three overlapping transcripts (3.6, 2.7 and 1.4 kb) encode four putative ORFs, UL133, UL135 and UL136 upstream of UL138, by canonical and stress-inducible alternative mechanisms of translation initiation (21). We hypothesized that UL135 and UL136 would be more detectable than UL133 transcripts, however, they exhibited a lower detection rate than UL133. In addition, the expression of UL135 and UL136 was not detected in the adjacent normal gastric tissues. The pattern of UL133 + UL138 exhibited the largest proportion in cancer tissues. A total of 69.64% (35/58) of UL138-positive cases were also detected in the UL133 transcripts of the gastric cancer tissues, while the expression in the normal tissues was 44.44% (24/54; P=0.008). In the corresponding normal tissues, the pattern of UL138 alone accounted for the majority of the expression patterns detected, which may indicate low levels or no HCMV latent infection in the majority of the normal tissues. Previous studies have revealed an association between the UL133 and UL138 proteins in fibroblasts, and the results of the present study further confirmed the interaction between UL133 and UL138 in gastric cancer tissues (19,20). Furthermore, the analysis of the HCMV gene transcription patterns may improve our understanding of the association between HCMV infection and gastric cancer.

## Figures and Tables

**Table I:** Primers and amplification conditions for PCR.

	Primers (5′ to 3′)		PCR conditions			
			
Name	Forward	Reverse	Annealing temperature, °C	Annealing time, sec	Cycles, n	Size, bp
UL133	TACCTGCCGATGGGTTCGCTACT	GGTTTGTCTTTCGCCCTACCTTTCTT	65	30	38	324
UL135	ATGGTGTGGCTGTGGCTCGGCGTCGGGCTCCTCG^a^	TCAGGTCATCTGCATTGACTCGGCGTCCTTCATG^a^	65	30	35	927
	GGATGGTCTGCCGATAGATAAACCCG^b^	CGCTGGCCGAGGACGACAAAGA^b^	57	30	35	143
UL136	ATGTCAGTCAAGGGCGTGGAGATGC^a^	TTACGTAGCGGGAGATACGGCGTTC^a^	60	30	35	723
	GCGGTGTTTCACGTTATCTGTGC^b^	ATGGCTCGCCGTCTGCTTCT^b^	65	30	35	191
UL138	ATGGACGATCTGCCGCTGAA^a^	TCACGTGTATTCTTGATGAT^a^	57	30	35	510
	GCTTACCACTGGCACGACACCT^b^	TACTCCCCGTACAGCTCGCAAC^b^	57	30	35	89
IE1	AGCCTTCCCTAAGACCACCAAT	CATAGCAGCACAGCACCCGACA	60	30	32	290

a Stage 1 and

b stage 2 primers used for nested PCR.

PCR, polymerase chain reaction; UL, unqiue long; IE, immediate-early.

**Table II:** Clinicopathological features and HCMV mRNA expression of 60 patients.

	UL133			UL135			UL136			UL138			IE1		
					
Clinicopathological features	Tumor, n	Normal, n	P-value	Tumor, n	Normal, n	P-value	Tumor, n	Normal, n	P-value	Tumor, n	Normal, n	P-value	Tumor, n	Normal, n	P-value
Gender			0.784			-			-			0.947			0.462
Male	26	18		3	0		5	0		37	36		9	5	
Female	15	9		3	0		2	0		19	18		6	6	
Age, years			0.869			-			-			0.881			0.428^a^
<60	16	10		1	0		3	0		21	21		7	3	
≥60	25	17		5	0		4	0		35	33		8	8	
Tumor size, cm			0.884			-			-			0.978			0.951
<5	22	14		3	0		2	0		32	31		7	5	
≥5	19	13		3	0		5	0		24	23		8	6	
Differentiation			0.500			-			-			0.731			0.426^a^
Well/moderate	17	9		0	0		0	0		20	21		5	6	
Poor/none	24	18		6	0		7	0		36	33		10	5	
TNM stage			0.698^a^			-			-			0.791			1.000^a^
I	6	6		0	0		0	0		11	8		0	0	
II	12	6		1	0		0	0		15	16		6	4	
III	23	15		5	0		7	0		30	30		9	7	
IV	0	0		0	0		0	0		0	0		0	0	

a Calculated using Fisher’s exact test.

UL, unique long; IE, immediate-early; TNM, tumor-node-metastasis; HCMV, human cytomegalovirus.

**Table III:** Analysis of the different transcription patterns of the UL133–138 locus and the clinicopathological features of the normal and tumor tissues.

			Pathological differentiation, n				TNM stage, n				Diameter of tumor (cm), n	
					
Tissue	Patterns of infection	Count	P	M-P	M	W	IV	III	II	I	Large (≥5)	Small (<5)
Tumor												
	UL133	2	1	0	1	0	0	1	1	0	2	0
	UL138	11	5	2	4	0	0	3	3	5	5	6
	UL133 + UL138	35	15	4	9	7	0	18	11	6	14	21
	UL135 + UL138	2	2	0	0	0	0	2	0	0	0	2
	UL136 + UL138	4	3	1	0	0	0	3	1	0	2	2
	UL133 + UL135 + UL138	1	1	0	0	0	0	1	0	0	0	1
	UL133 + UL135 + UL136 + UL138	3	2	1	0	0	0	3	0	0	3	0
Normal												
	UL133	3	2	0	1	0	0	3	0	0	3	0
	UL138	30	8	9	8	5	0	18	10	2	13	17
	UL133 + UL138	24	13	3	5	3	0	12	6	6	10	14

P, poor; M-P, moderate-poor; M, moderate; W, well. UL, unique long; TNM, tumor-node-metastasis.
